# **Baseline abundance of oxalate-degrading bacteria determines response to** ***Oxalobacter formigenes*** **probiotic therapy**

**DOI:** 10.1080/19490976.2025.2562337

**Published:** 2025-09-23

**Authors:** Mangesh Suryavanshi, Arthur Franklin, Sonia Fargue, Dean G. Assimos, John Knight, Aaron W. Miller

**Affiliations:** a Department of Cardiovascular and Metabolic Sciences, Cleveland Clinic, Cleveland, OH, USA; b Department of Urology, University of Alabama at Birmingham, Birmingham, AL, USA; c Department of Urology, Cleveland Clinic, Cleveland, OH, USA

**Keywords:** Oxalate, hyperoxaluria, *Oxalobacter formigenes*, oxalate-degrading bacteria, probiotics

## Abstract

Oxalate, a compound derived from both diet and metabolism, contributes to multiple renal and vascular diseases. Certain gut bacteria degrade oxalate, limiting absorption and promoting secretion. This study examined microbial factors that influence the effectiveness of *Oxalobacter formigenes*, a specialized oxalate-degrading bacterium, in lowering urinary oxalate levels. We analyzed gut microbiota from a controlled diet study involving 26 healthy, non–stone-forming adults who were initially uncolonized and then colonized with *O. formigenes*. Stool samples were profiled for 16S rRNA and oxalate-degrading genes—oxalyl-CoA decarboxylase (*oxc*) and formyl-CoA transferase (*frc*)—using high-throughput amplicon sequencing and qPCR. Comparative analyses assessed associations between microbial features and oxalate homeostasis, including changes in urinary oxalate excretion. The baseline abundance of oxalate-degrading genes (*oxc* and *frc*) was significantly and negatively correlated with stool oxalate (R = –0.43 for *frc*, –0.34 for *oxc*), urinary oxalate levels (R = –0.25 for *frc*, –0.27 for *oxc*), and the reduction in urine oxalate after *O. formigenes* administration (R = –0.36 for *frc*, –0.42 for *oxc*). This study provides the first direct evidence that baseline oxalate-degrading gene abundance predicts probiotic response. Results explain inconsistent clinical trial results and support precision microbiome-based therapy for hyperoxaluria via targeted patient stratification.

## Introduction

Oxalate is a compound found in plant-based foods and produced endogenously in the liver.^1^ Elevated oxalate levels are linked to chronic diseases such as urinary stone formation (USD),^2^ chronic kidney disease (CKD),^3^ end-stage renal disease (ESRD), along with vascular calcification and cardio-kidney-metabolic syndrome (CKMS).^4-6^ Hyperoxaluria, typically defined as urinary oxalate excretion above 40 mg/d in adults,^7^ increases disease risk, though lower levels of excretion may be linked to progression of CKD.^8^ These oxalate-related conditions impose a substantial global health and economic burden,^9-12^ highlighting the need for strategies to reduce urinary oxalate excretion.

Oxalate is abundant in plant-based foods such as leafy greens, nuts, seeds, beets, rhubarb, and certain grains,^13^^,^^14^ and when ingested in large quantities can result in kidney injury due to the formation and deposition of calcium oxalate crystals.^15^ Endogenous oxalate arises mainly from glyoxylate metabolism and ascorbic acid breakdown.^16^^,^^17^ Because mammals cannot degrade oxalate,^18^ it must be eliminated via urinary excretion or by degradation in the gut by specific bacteria,^19-23^ and thus may mitigate the risk of oxalate-associated diseases.

Gut bacteria employ multiple metabolic pathways for oxalate degradation. One of the most prominent pathways involves the *oxlT* gene, which encodes an oxalate-formate antiporter protein,^24^ which is often coupled with the *frc* and *oxc* genes, encoding formyl-CoA transferase and oxalyl-CoA decarboxylase, respectively. These enzymes sequentially convert oxalate to formyl-CoA and oxalyl-CoA, and subsequently to CO₂ and formyl-CoA.^25^^,^^26^

*Oxalobacter formigenes*, identified over 45 y ago, is the canonical oxalate-degrading bacterium and considered the most effective oxalate-degrading species.^27^ However, results from clinical intervention trials using this species have varied widely, calling into question the factors that determine success.^28-30^ This inconsistency highlights the need to identify microbial or host factors that predict therapeutic response. In our recent clinical trial involving healthy adults not colonized with *O. formigenes*, a single ingestion of viable *O. formigenes* cells resulted in successful colonization in all participants.^30^ However, while overall urinary oxalate levels decreased significantly following colonization, individual responses varied considerably from an increase of 5 mg/day of urinary oxalate to a decrease of > 15 mg/d,^30^ similar to past clinical intervention studies with this species.^28^ Therefore, the aim of this study was to determine the specific microbial factors that predict the magnitude of response to *O. formigenes* probiotic therapy in terms of urinary oxalate reduction, focusing on other oxalate-degrading bacteria. We hypothesized that the baseline presence of other oxalate-degrading bacteria influences the efficacy of the *O. formigenes* probiotic.

## Materials and methods

### Study design and participant recruitment

In the original sequential, diet-controlled clinical trial (ClinicalTrials.gov NCT03752684),^30^ participant screening included serum metabolic panels, and urine creatinine (two 24-h collections), to confirm eligibility and data reliability. Exclusion criteria included abnormal lab values, recent antibiotic use, kidney stone history, and extremes in BMI or renal function. Those who passed initial health screens, were further screened for *O. formigenes* colonization using stool culture. Specifically, fresh stool samples were inoculated into Schaedler anaerobe broth supplemented with 20 mM sodium oxalate and incubated anaerobically at 37 °C for 5–7 d. Colonies were sub-cultured on oxalate-containing agar and verified using *O. formigenes*-specific PCR primers, as previously described.^22^^,^^31^ Participants confirmed to be non-colonized after two assessments—one on a self-selected diet and another on an oxalate-enriched diet—were enrolled.

Enrolled participants followed a structured dietary protocol: a low-oxalate (~50 mg/d), normal-calcium (~1200 mg/d) diet; a ≥ 2-week washout on a self-selected diet; then a high-oxalate (~230 mg/d), low-calcium (~600 mg/d) diet. Colonization with *O. formigenes* was induced by oral ingestion of ~10¹⁰ live cells. Stool cultures confirmed absence of colonization before and successful colonization after inoculation. Stool and 24-h urine collections were performed on days 3–5 of each dietary phase to allow for equilibration. Colonization persistence was assessed by stool culture during follow-up (up to 4 y).

### Controlled dietary interventions

Prior to induction of *O. formigenes* colonization, participants collected stool and 24-h urine specimens while consuming standardized low-oxalate (50–60 mg/d) and high-oxalate (210–240 mg/d) diets. The differences in urine oxalate excretion and dietary oxalate content of the high and low oxalate diets was used to estimate dietary oxalate absorption. Following induction of *O. formigenes* colonization, stool and 24-h urine were collected only on the high oxalate diet to assess the efficacy of colonization for reducing urine oxalate. Oxalate levels from pre- and post-colonization urine samples on the high oxalate diet were compared as this dietary phase provided a strong and consistent oxalate challenge across participants to evaluate degradation capacity. Oxalate levels were measured by ion chromatography-mass spectrometry (IC-MS).

### Stool sample collection and DNA extraction

Microbiome analyses were only performed on multiple stool samples collected for each patient prior to *O. formigenes* colonization. Stool samples were collected in sterile containers and immediately frozen at −80 °C. Stool DNA was extracted through a semi-automated protocol on a KingFisher Duo Prime System (Thermo Scientific) following the manufacturer’s protocol for stool. A total of 224 stool samples were collected from the 22 patients, with 122 samples from participants on a high oxalate diet and 102 samples from a low oxalate diet.

### High throughput sequencing

The V4 region of the 16S rRNA gene was amplified using the 515F and 806 R bacterial primers. To quantify oxalate metabolism potential, high throughput formyl-CoA transferase (*frc*) gene sequencing was performed using conserved, validated primers: frc-171F (5ʹ-CTSTAYTTCACSATGCTSAAC−3′) and frc-306R (5′-GDSAAGCCCATVCGRTC−3′). Amplicons were sequenced on the Illumina MiSeq (2 × 250 bp paired-end reads). Raw sequences were processed using DADA2 (version 1.18)^32^ for quality control and amplicon sequence variant (ASV) assignment. Taxonomy and gene annotation was assigned using a non-redundant database of the SILVA 138 SSURef and NCBI 16S rRNA database for 16S rRNA sequences,^33^ and to the UniRef90 database for *frc* sequences. Taxa assigned to mitochondria or chloroplasts (16S rRNA gene) or that were not annotated to *frc (frc* gene) were removed. The resulting ASV’s were aligned in MSA and arranged into a maximum likelihood phylogeny in phangorn.^34^ Taxonomic annotation of the *frc* sequences was conducted by mapping reads to a reference dataset of 45,000 + full-length prokaryotic genomes from NCBI, with BWA.^35^ To evaluate redundancy and compare taxonomy from the 16S rRNA and *frc* genes, ASV’s were clustered at 97% similarity using the CD-HIT algorithm. Alpha and beta diversity metrics were calculated using phylogenetic diversity metrics.

### qPCR of oxalate-degrading genes

The abundance of all bacteria and the oxalate-degrading fraction was quantified by qPCR of the *16S rRNA,* and oxalyl-CoA decarboxylase *(oxc*) and *frc* genes, respectively. Primer sequences, amplicon size and standard bacteria used for quantification have been previously reported.^30^ These primers broadly target the *oxc* and *frc* genes from diverse taxonomic backgrounds.^36^^,^^37^ Triplicate qPCR reactions were setup (10 μl each) containing the appropriate pair of primers, 50 μg of metagenomic DNA and SYBR green master mix provided by Applied Biosystems Inc. (Thermo Fisher Scientific, USA). Reactions were run on StepOnePlus Real time PCR system from Applied Biosystems. Standard curves were generated from serial dilutions of a known concentration of PCR products derived from DNA of standard bacterial species. Melting curve analysis was performed at the end of qPCR cycles to validate amplification specificity. Average values of the triplicates were used for downstream analyses. For all assays, PCR efficiency was maintained above 90% with a correlation coefficient > 0.99. Data were normalized through log2 transformation of the proportional gene copy numbers of the *oxc* or *frc* genes relative to the copy numbers of the 16S rRNA gene to ensure that gene abundance data reflected biological differences rather than differences in microbial biomass.

### Statistical analysis

All statistical analyses were conducted using R (version 4.1.2). Pearson correlations were employed to assess impact of oxalate-degrading gene abundance on metrics of oxalate homeostasis (stool oxalate, 24-h urinary oxalate excretion and estimated gastrointestinal oxalate absorption), and the change in urinary oxalate excretion following induction of *O. formigenes* colonization on the high oxalate controlled diet. One or two-way ANOVA’s with Holm’s-corrected, post-hoc paired *t*-tests were employed to compare continuous variables, where applicable. For correlations, *t*-tests on the correlation coefficient were employed, where applicable.

To identify bacterial taxa that differed in abundance between study groups, differential abundance analysis was performed using the DESeq2 package.^38^ DESeq2 uses a negative binomial distribution to account for variability in microbial sequencing data and applies internal normalization to correct for differences in library size. Taxa with a false discovery rate (FDR)-adjusted *p*-value < 0.05 were considered statistically significant. Alpha diversity, calculated as an unweighted Phylogenetic Diversity, quantifies the number of unique phylogenetic clades between groups.^39^ Alpha diversity comparisons were conducted using Holm’s corrected, paired *t*-tests. Beta diversity differences, which quantifies microbiome composition between groups based on the presence–absence and relative abundance of ASVs, were assessed using permutational multivariate analysis of variance (PERMANOVA) with weighted Unifrac dissimilarity matrices.^40^^,^^41^ The Unifrac metric quantifies differences based on phylogenetic clades. The PERMANOVA statistical test is a non-parametric test determines whether microbial communities differ significantly across groups based on their overall structure. Holm’s corrected *p* values of < 0.05 were considered statistically significant.

## Results

### Results of patient screening

Of the 38 individuals initially assessed for eligibility, 12 were excluded due to recent antibiotic use (*n* = 5), history of nephrolithiasis (*n* = 3), abnormal serum or urinary markers (*n* = 2), or BMI outside the inclusion range (*n* = 2). The final cohort included 26 healthy adults who met all eligibility criteria, with baseline laboratory values within reference ranges. However, one participant dropped out after successful colonization, but before the final dietary phase. Three participants withdrew prior to colonization. Twenty-two participants completed the trial.

### Dietary oxalate intake strongly influences urinary oxalate excretion

As previously reported, participants consuming a high-oxalate diet exhibited significantly increased oxalate in both stool and urine compared to the low-oxalate diet.^30^

These data are consistent with an earlier report by Holmes and colleagues^42^ and confirms that dietary oxalate is a major contributor to urinary oxalate levels and validates the model for testing probiotic interventions.

### Baseline abundance of oxalate-degrading genes negatively correlates with stool and urine oxalate levels and the response to *O. formigenes* colonization

**Figure 1.**
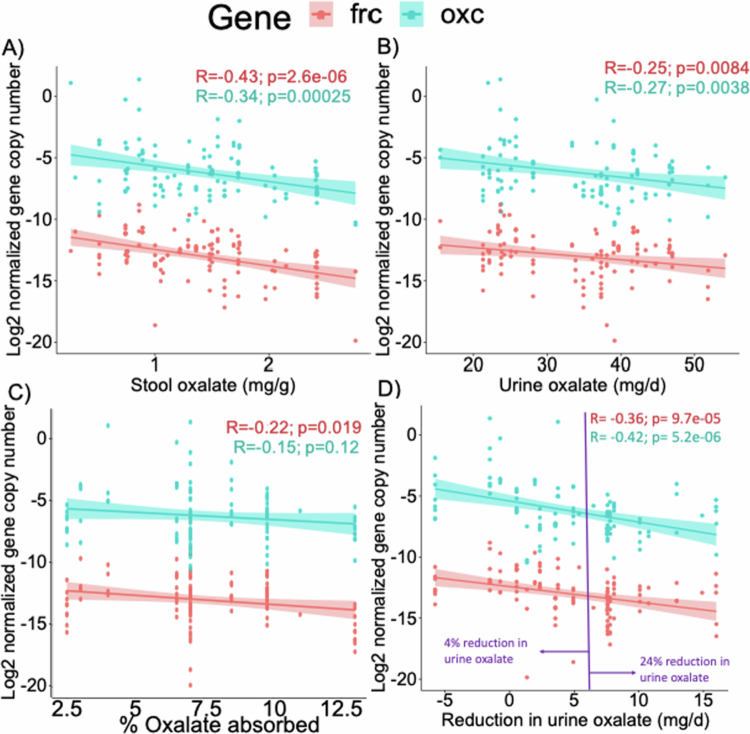
Oxalate-degrading gene abundance influences oxalate metrics and response to *O. formigenes* colonization. (A–D) *frc* and *oxc* gene abundance correlated to stool oxalate (A), urine oxalate (B), percent estimated dietary oxalate absorption (C), and reduction in urine oxalate after *O. formigenes* colonization (D). Gene copy numbers were Log2 transformed and normalized to 16S rRNA gene copy number. Purple line divides data into lower and upper halves of response. The % reduction in urine oxalate for each half is listed. *p* values are based on *t*-tests for Pearson’s correlation coefficient.

To assess the impact of the baseline abundance of oxalate-degrading bacteria on stool oxalate concentration, urinary oxalate excretion and estimated gastrointestinal oxalate absorption, we quantified the pre-colonization abundance of *oxc* and *frc genes*. There were significant negative correlations between the baseline abundance of oxalate-degrading genes with stool oxalate concentrations (R = −0.43, *p *< 0.001 for *frc* and R = −0.34, *p *< 0.001 for *oxc*; Figure 1A), urine oxalate excretion (R = −0.25, *p* = 0.008 for *frc* and R = −0.27, *p* = 0.004 for *oxc*; Figure 1B), estimated gastrointestinal oxalate absorption (R = −0.22, *p* = 0.019 for *frc*; Figure 1C), and reduction in urine oxalate after induction of *O. formigenes* colonization (R = −0.36, *p *< 0.001 for *frc* and R = −0.42, *p *< 0.001 for *oxc*; Figure 1D); *oxc* abundance and estimated gastrointestinal oxalate absorption produced a non-significant correlation (R = −0.15, *p* = 0.12 for *oxc*; Figure 1C). Among the 11 participants with the least reduction in urinary oxalate (*n* = 124 samples), the average decrease was only 4%. In contrast, the top 11 responders (*n* = 100 samples) saw a 24% reduction, highlighting the high degree of variability in response (Figure 1D).

### Diversity of frc-containing bacteria and the whole gut microbiota

**Figure 2.**
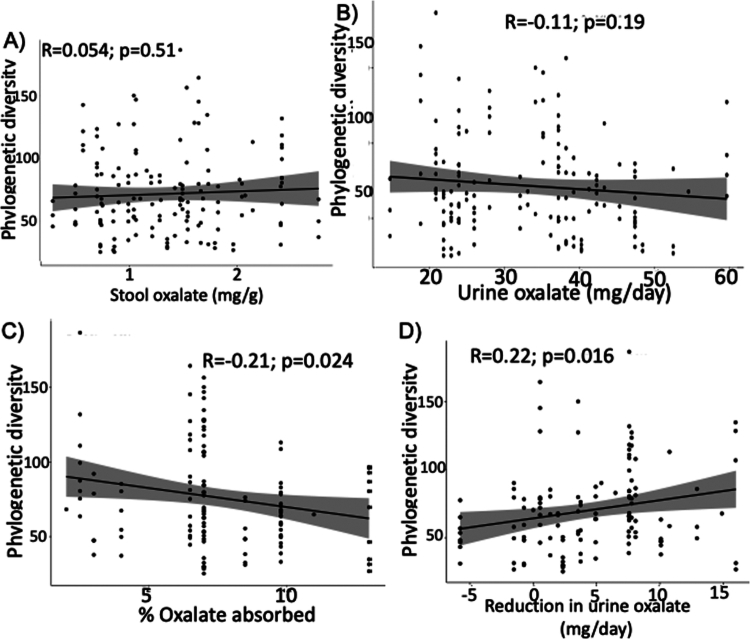
Number of phylogenetic groups in the *frc-*containing fraction of the gut microbiota is associated with metrics for oxalate homeostasis and response to *O. formigenes* colonization. (A–D) Number of phylogenetic groups correlated to stool oxalate (A), urine oxalate (B), percent estimated dietary oxalate absorption (C), and reduction in urine oxalate after *O. formigenes* colonization. Metric was quantified as phylogenetic diversity, based on high throughput sequencing of the frc gene. *p* values are based on *t*-tests for Pearson’s correlation coefficient.

**Figure 3.**
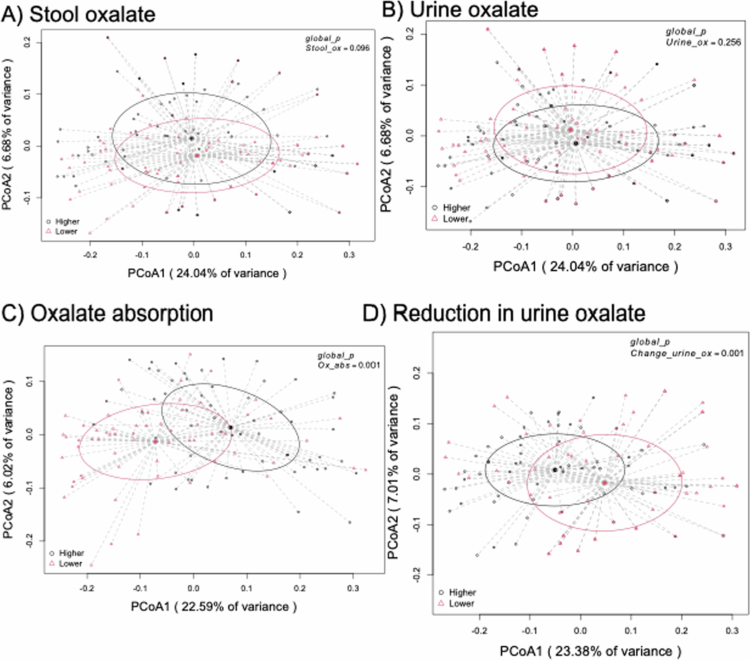
Composition of the *frc-*containing fraction of the gut microbiota is associated with metrics for oxalate homeostasis and response to *O. formigenes* colonization. (A–D) Composition associated with stool oxalate (A), urine oxalate (B), percent estimated dietary oxalate absorption (C), and reduction in urine oxalate after *O. formigenes* colonization. Metric was quantified as a weighted UniFrac dissimilarity matrix, visualized through PCoA plots, based on high throughput sequencing of the frc gene. The continuous variables were converted to binary categorical variables based on values that were in the higher or lower half of all values. *p* values are based on PERMANOVA with 999 permutations.

Taxonomic profiling revealed that bacteria containing the oxalate-degrading gene *frc* form a distinct taxonomic profile (Figure S1). *Nonomuraea*, *Pseudoduganella*, and *Streptomyces* dominated the frc-containing taxa. When comparing the number of unique sequences at 97% homology for the *frc* gene to 16S rRNA gene, we estimated that 5.7% of all taxa in the gut contain an *frc* gene. There was no significant correlation between the number of *frc* phylogenetic clades and stool oxalate (R = 0.054, *p* = 0.51; Figure 2A) nor urine oxalate (R = −0.11, *p* = 0.19; Figure 2B). However, there were significant correlations between the number of *frc* phylogenetic clades and estimated gastrointestinal oxalate absorption (R = −0.21, *p* = 0.024; Figure 2C), and response to *O. formigenes* colonization (R = 0.22, *p* = 0.016; Figure 2D). These data are consistent with the association between the composition of the *frc-*fraction of the gut microbiota and oxalate metrics. Specifically, there was no association with stool oxalate (*p* = 0.096; Figure 3A) nor urine oxalate (*p* = 0.256; Figure 3B), but a significant association for estimated gastrointestinal oxalate absorption (*p* = 0.001; Figure 3C) and response to *O. formigenes* colonization (*p* = 0.001; Figure 3D).

In contrast to the association between *frc* diversity and oxalate metrics, when looking at the 16S rRNA gene with high throughput sequencing, we found that the number of phylogenetic clades exhibited a significant, positive correlation to stool oxalate (R = 0.33, *p *< 0.001; Figure S2A), with no correlations to urine oxalate (R = 0.12, *p* = 0.16; Figure S2B), estimated gastrointestinal oxalate absorption (R = −0.16, *p* = 0.094; Figure S2C), nor response to *O. formigenes* (R = −0.016, *p* = 0.87; Figure S2D). However, there was a strong association in the total composition of the gut microbiota and all oxalate metrics (*p* = 0.001 for all metrics; Figures S3A–D).

**Figure 4.**
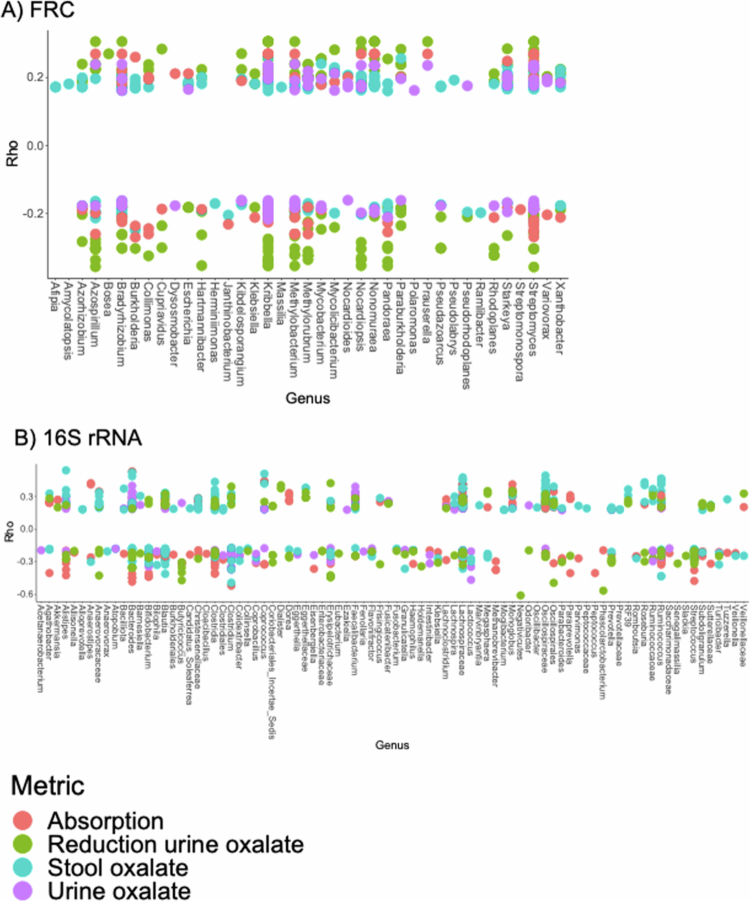
Spearman correlations between specific ASV’s and metrics for oxalate homeostasis, based on the *frc* (A) or 16S rRNA (B) genes. Each dot represents a single ASV, colored by the specific metric of correlation. Only significant correlations are shown, based on FDR-corrected Spearman correlations between ASV abundance and the oxalate homeostasis metrics.

Spearman correlations between oxalate metrics and the *frc* or 16S rRNA ASVs identified multiple taxa that exhibited positive or negative associations with these metrics. Specifically, among *frc-*taxa, the *Bradyrhizobium, Kribbella, Methylobacterium,* and *Streptomyces* had the most ASVs significantly associated to oxalate metrics (FDR < 0.05; Figure 4A). Among all taxa, the *Alistiipes, Bacteriodes, Lachnospiraceae, Oscillospiraceae,* and *Ruminococcus* were the most associated with oxalate metrics (Figure 4B), consistent with past reports.^28^^,^^43^

## Discussion

Animal studies and surveys of human populations have identified diverse oxalate-degrading bacteria.^43-49^ It is estimated that 35% of all species in the human gut contains at least one oxalate-degrading gene,^47^ while 60% of species harbor oxalate-degrading genes in oxalate-consuming rodents,^43^ suggesting that the diversity of oxalate-degrading bacteria plays an important role in oxalate homeostasis, particularly when dietary oxalate intake is high. Here, we found that 6% of species harbored the *frc* gene, which is just one of many known oxalate-degrading genes.^47^ In this controlled diet study, we show that individuals with lower baseline levels of oxalate-degrading bacteria experienced greater benefit from *O. formigenes* colonization, as measured by reduced urinary oxalate. Oxalate-degrading gene abundance was significantly associated with stool and urine oxalate levels, as well as with gastrointestinal oxalate absorption, underscoring the central role of microbial gene abundance in regulating oxalate homeostasis. In contrast, the diversity of oxalate-degrading bacteria was less strongly linked to these outcomes and to the efficacy of *O. formigenes* colonization. Specifically, the lack of correlation between phylogenetic diversity of *frc*-containing taxa and stool or urinary oxalate suggests that the oxalate-degrading niche is neither dominated by a few functionally superior species (which would produce a positive correlation) nor broadly saturated by metabolically diverse taxa (which would result in a negative correlation). However, the observed associations between *frc* gene phylogenetic diversity and both oxalate absorption and the probiotic response to *O. formigenes* imply that a subset of dominant taxa may still exert meaningful influence on niche occupancy and therapeutic outcomes. Future studies should aim to disentangle the relative contributions of gene abundance and community composition to oxalate metabolism and probiotic efficacy.

These findings, particularly in relation to oxalate-degrading gene abundance, may explain the inconsistent results from prior probiotic trials and suggest a clear microbial biomarker for patient stratification.^28-30^ Interestingly, the results of this human study, which show that *O. formigenes* is only effective when there is a low baseline level of oxalate-degrading bacteria, mirror findings from our wild rodent studies,^43^ indicative of the robustness of these results and highlights the value of studying oxalate degradation in animal models consuming a naturally high oxalate containing diet.

A prior study evaluated 24-h stool oxalate levels in healthy individuals not colonized with *O. formigenes* while on a controlled diet (250 mg oxalate, 400 mg calcium), similar to the study design presented here.^31^ The authors found that, on average, ~80% of ingested oxalate was recovered in stool, suggesting ~20% intestinal degradation even in the absence of *O. formigenes*. Notably, there was substantial inter-individual variability, with oxalate degradation ranging from 0% to 40%. Although stool samples were not used for microbial analysis, these findings suggest that there is considerable inter-individual variability in oxalate-degrading bacteria and that these bacteria may compensate for the absence of *O. formigenes,* similar to the study results presented here.

The gut microbiome influences all facets of human health and physiology.^50^^,^^51^ Despite advances in understanding the specific mechanisms through which gut bacteria influence host health,^51^ clinical probiotic trials consistently exhibit a high degree of variability in patient response.^52-56^ The results reported here support the need to incorporate profiling the abundance of background oxalate-degrading genes into patient selection criteria for future clinical trials assessing intestinal oxalate degradation therapies and supports similar approaches for other probiotic studies.

The question remains whether *O. formigenes* alone is the optimal therapeutic agent for reducing urinary oxalate excretion, even when accounting for the baseline abundance of oxalate-degrading bacteria. Differences in beta-diversity metrics between the *frc*-containing and total microbial communities suggest that other bacterial functions, such as short-chain fatty acid (SCFA) production and bile acid metabolism, may indirectly influence oxalate homeostasis.^43^^,^^57^^,^^58^ ​​​​Future research should explore combination therapies such as co-administering *O. formigenes* with other oxalate-degrading bacteria, SCFA producers, or bile acid-modulating microbes, particularly in individuals with low oxalate-degrading bacterial abundance.

Limitations of the study include the small cohort consisting of healthy adults using gene abundance and clinically relevant oxalate parameters as the inputs and outcomes. *In vitro* quantification of oxalate degradation from participant stool would have increased mechanistic insight. However, multiple samples were collected longitudinally across controlled low and high oxalate diets, increasing the robustness of the findings here. Additionally, data recapitulate findings in robust preclinical studies that involved microbiological, and targeted microbial transplant studies, further increasing the validity of the current findings.^43^ While the focus on healthy patients allowed for a controlled evaluation of microbial factors involved in oxalate handling and response to *O. formigenes* probiotics, further studies in patients with primary or secondary hyperoxaluria, or impaired renal function, are warranted to validate the predictive utility of *oxc* and *frc* gene abundance in clinical populations. Another limitation is that we profiled only two oxalate-degrading genes (*frc* and *oxc*), despite the existence of additional oxalate-degrading genes, which may have led to an underestimation of the baseline abundance and functional potential of oxalate-degrading bacteria in the gut microbiome. Although diet and medication use were strictly controlled through study design and pre-screening, we did not assess host genetic factors such as SLC26A1 or SLC26A6 polymorphisms, which may influence intestinal oxalate transport and urinary excretion. Future studies integrating host genetic profiling with microbiome and metabolomic analyses could provide a more comprehensive understanding of inter-individual variation in oxalate homeostasis. Finally, microbiome profiling in this study was performed on samples prior to *O. formigenes* colonization to prospectively assess predictive relationships between baseline microbial features and therapeutic response. While this design addresses our primary objective, it does not capture post-colonization shifts in microbial community structure. On-going analyses of longitudinal microbiome samples from this cohort will help elucidate ecological changes and host–microbe interactions that may further modulate probiotic efficacy. Future studies are needed to determine the impact on gut microbiota after induction of *O. formigenes* colonization, to determine the relationship between oxalate-degrading gene abundance and functional oxalate-degrading capacity, and to examine other microbial functions beyond oxalate degradation that may influence oxalate homeostasis.

## Conclusions

Hyperoxaluria is associated with multiple chronic disorders that account for substantial mortality and morbidity. Such individuals could potentially benefit from oxalate-degrading therapies, including *O. formigenes* colonization. Unfortunately, only 50% of clinical intervention studies with this species have shown success.^28-30^ Our findings indicate that baseline levels of oxalate-degrading bacteria influence the effectiveness of *O. formigenes* probiotic therapy. This finding offers a clear explanation for previously inconsistent results and underscores the importance of quantitatively assessing *oxc* and *frc* gene abundance—beyond simply detecting *O. formigenes*—to identify patients most likely to benefit from oxalate-lowering therapies.

## Acknowledgments

We thank Tamara Keenum and Michelle Bui for their technical assistance and Demond Wiley for coordinating study recruitment and retention. We greatly appreciate the help of the Clinical Research Units at UAB (supported by NIH grant UL1TR003096). We appreciate the help of Sromona Mukherjee in uploading sequencing data to the sequence read archive. This work was funded by NIH grants R01DK087967, R03DK129497, K08DK115833, R01DK126774, R01DK128160, UL1TR003096, R01DK121689. ClinicalTrials.gov identifier: NCT03752684.

## Author contributions

JK, DGA, and SF designed the original clinical study. MS, AWM, and JK designed the current study. MS conducted the experiments. AWM, AF, and MS analyzed the data. All authors drafted, revised, and approved the final version of the manuscript.

## Disclosure of potential conflicts of interest

No potential conflicts of interest were disclosed.

## Supplementary Material

Supplemental Figures **Figure S1**. Genus-level taxonomic distribution of the whole gut microbiota, based on the 16S rRNA gene, and the frc-containing fraction.
**Figure S2**. Number of phylogenetic groups in whole gut microbiota mildly associated with metrics for oxalate homeostasis and response to O. formigenes colonization. A-D) Number of phylogenetic groups correlated to stool oxalate (A), urine oxalate (B), percent estimated dietary oxalate absorption (C), and reduction in urine oxalate after O. formigenes colonization. Metric was quantified as phylogenetic diversity, based on high throughput sequencing of the 16S rRNA gene. P-values are based on t-tests for Pearson’s correlation coefficient.
**Figure S3**. Composition of the whole gut microbiota is associated with metrics for oxalate homeostasis and response to O. formigenes colonization. A-D) Composition associated with stool oxalate (A), urine oxalate (B), percent estimated dietary oxalate absorption (C), and reduction in urine oxalate after O. formigenes colonization. Metric was quantified as a weighted UniFrac dissimilarity matrix, visualized through PCoA plots, based on high throughput sequencing of the 16S rRNA gene. The continuous variables were converted to binary categorical variables based on values that were in the higher or lower half of all values. P-values are based on PERMANOVA with 999 permutations.

## Data Availability

Raw high throughput sequencing data are available at the sequence read archive through Project number PRJNA1051530. Data can be found at https://www.ncbi.nlm.nih.gov/bioproject/PRJNA1051530.
