# Supplementary material for: Baseline abundance of oxalate-degrading bacteria determines response to Oxalobacter formigenes probiotic therapy
Source: Gut Microbes. 2025 Sep 23;17(1):2562337. doi: 10.1080/19490976.2025.2562337 (PMC12459359; doi:10.1080/19490976.2025.2562337)
Supplement: Supplemental Figures [file KGMI_A_2562337_SM4178.pdf]

Supplemental Figures

Figure S1. Genus-level taxonomic distribution of the whole gut microbiota, based on the 16S rRNA gene, and the *frc*-containing fraction.

Figure S2. Number of phylogenetic groups in whole gut microbiota mildly associated with metrics for oxalate homeostasis and response to *O. formigenes* colonization. A-D) Number of phylogenetic groups correlated to stool oxalate (A), urine oxalate (B), percent estimated dietary oxalate absorption (C), and reduction in urine oxalate after *O. formigenes* colonization. Metric was quantified as phylogenetic diversity, based on high throughput sequencing of the 16S rRNA gene. P-values are based on t-tests for Pearson's correlation coefficient.

Figure S3. Composition of the whole gut microbiota is associated with metrics for oxalate homeostasis and response to *O. formigenes* colonization. A-D) Composition associated with stool oxalate (A), urine oxalate (B), percent estimated dietary oxalate absorption (C), and reduction in urine oxalate after *O. formigenes* colonization. Metric was quantified as a weighted UniFrac dissimilarity matrix, visualized through PCoA plots, based on high throughput sequencing of the 16S rRNA gene. The continuous variables were converted to binary categorical variables based on values that were in the higher or lower half of all values. P-values are based on PERMANOVA with 999 permutations.

Figure S1

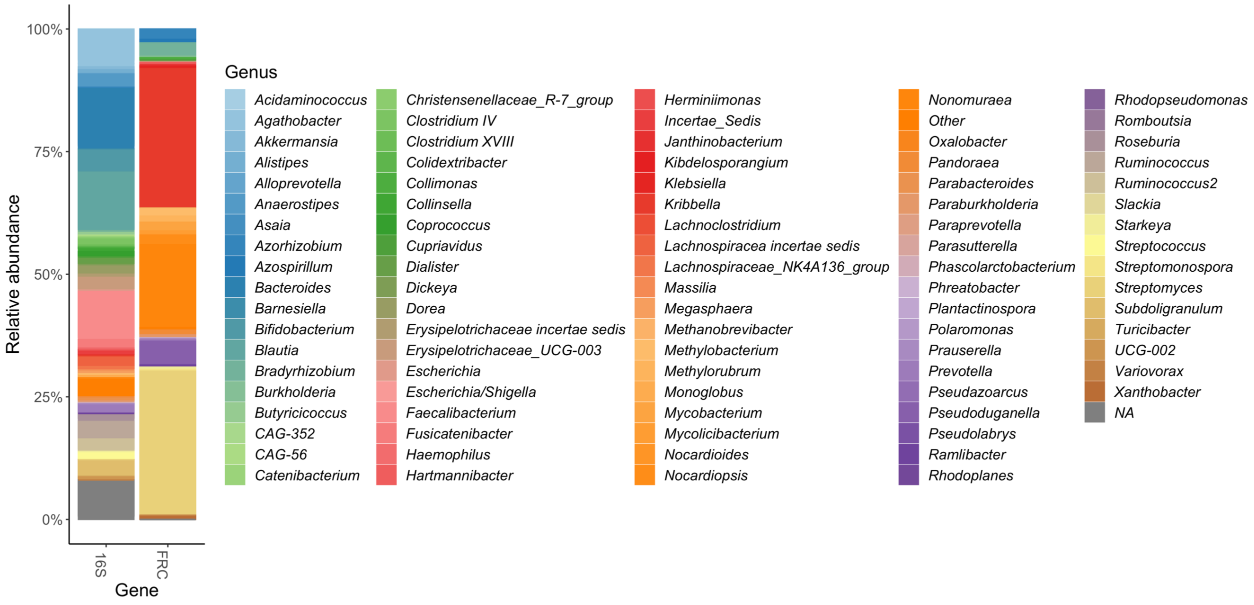

100%
75%
50%
25%
0%
Relative abundance
16S
FRC
Gene
Genus
Acidaminococcus
Agathobacter
Akkermansia
Alistipes
Alloprevotella
Anaerostipes
Asaia
Azorhizobium
Azospirillum
Bacteroides
Barnesiella
Bifidobacterium
Blautia
Bradyrhizobium
Burkholderia
Butyricicoccus
CAG-352
CAG-56
Catenibacterium
Christensenellaceae_R-7_group
Clostridium IV
Clostridium XVIII
Colidextribacter
Collimonas
Collinsella
Coprococcus
Cupriavidus
Dialister
Dickeya
Dorea
Erysipelotrichaceae incertae sedis
Erysipelotrichaceae_UCG-003
Escherichia
Escherichia/Shigella
Faecalibacterium
Fusicatenibacter
Haemophilus
Hartmannibacter
Herminiimonas
Incertae_Sedis
Janthinobacterium
Kibdelosporangium
Klebsiella
Kribbella
Lachnoclostridium
Lachnospiracea incertae sedis
Lachnospiraceae_NK4A136_group
Massilia
Megasphaera
Methanobrevibacter
Methylobacterium
Methylorubrum
Monoglobus
Mycobacterium
Mycolicibacterium
Nocardioides
Nocardiopsis
Nonomuraea
Other
Oxalobacter
Pandoraea
Parabacteroides
Paraburkholderia
Paraprevotella
Parasutterella
Phascolarctobacterium
Phreatobacter
Plantactinospora
Polaromonas
Prauserella
Prevotella
Pseudazoarcus
Pseudoduganella
Pseudolabrys
Ramlibacter
Rhodoplanes
Rhodopseudomonas
Romboutsia
Roseburia
Ruminococcus
Ruminococcus2
Slackia
Starkeya
Streptococcus
Streptomonospora
Streptomyces
Subdoligranulum
Turicibacter
UCG-002
Variovorax
Xanthobacter
NA

Figure S2

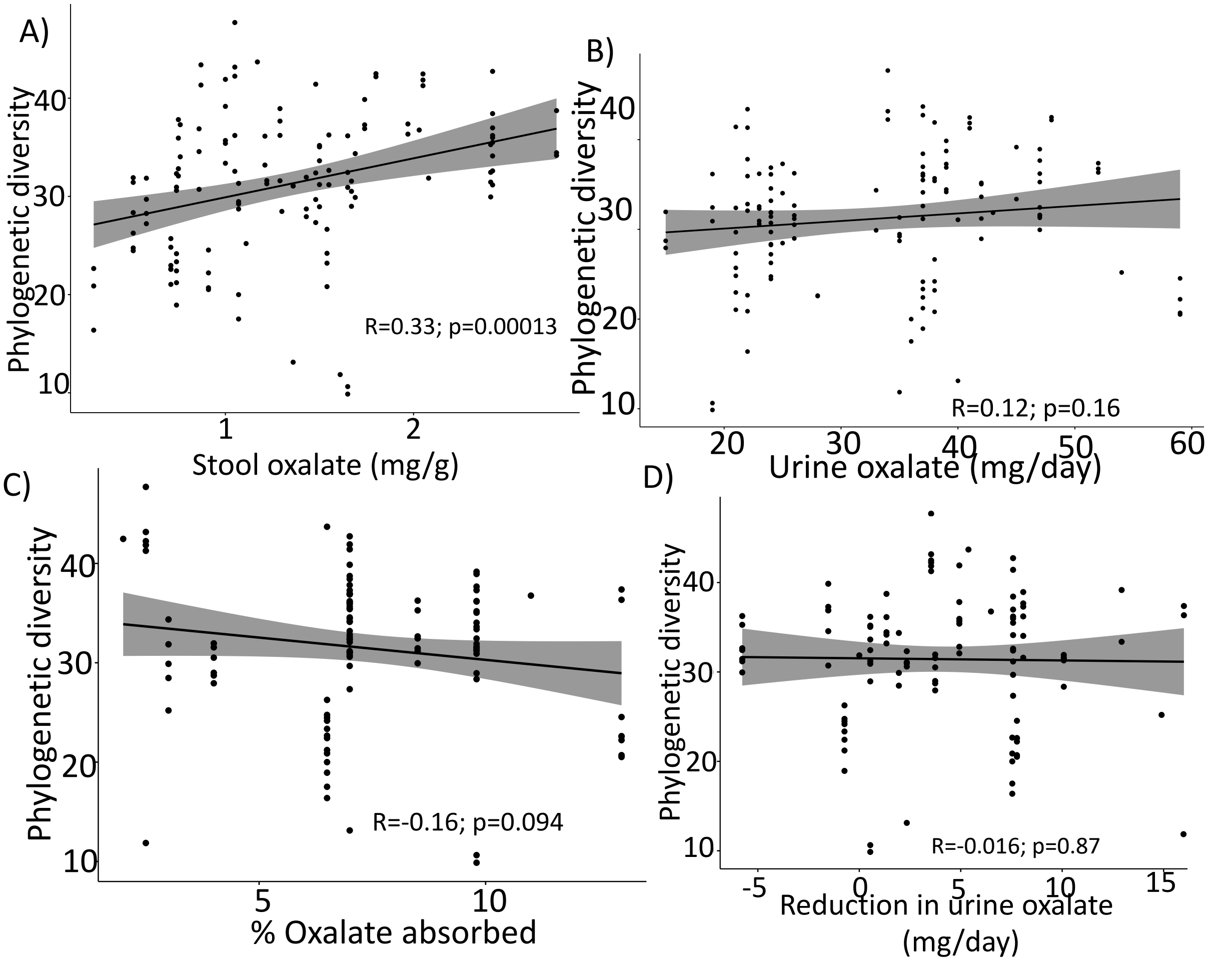
A)
Phylogenetic diversity
Stool oxalate (mg/g)
R=0.33; p=0.00013
B)
Phylogenetic diversity
Urine oxalate (mg/day)
R=0.12; p=0.16
C)
Phylogenetic diversity
% Oxalate absorbed
R=-0.16; p=0.094
D)
Phylogenetic diversity
Reduction in urine oxalate (mg/day)
R=-0.016; p=0.87

Figure S3

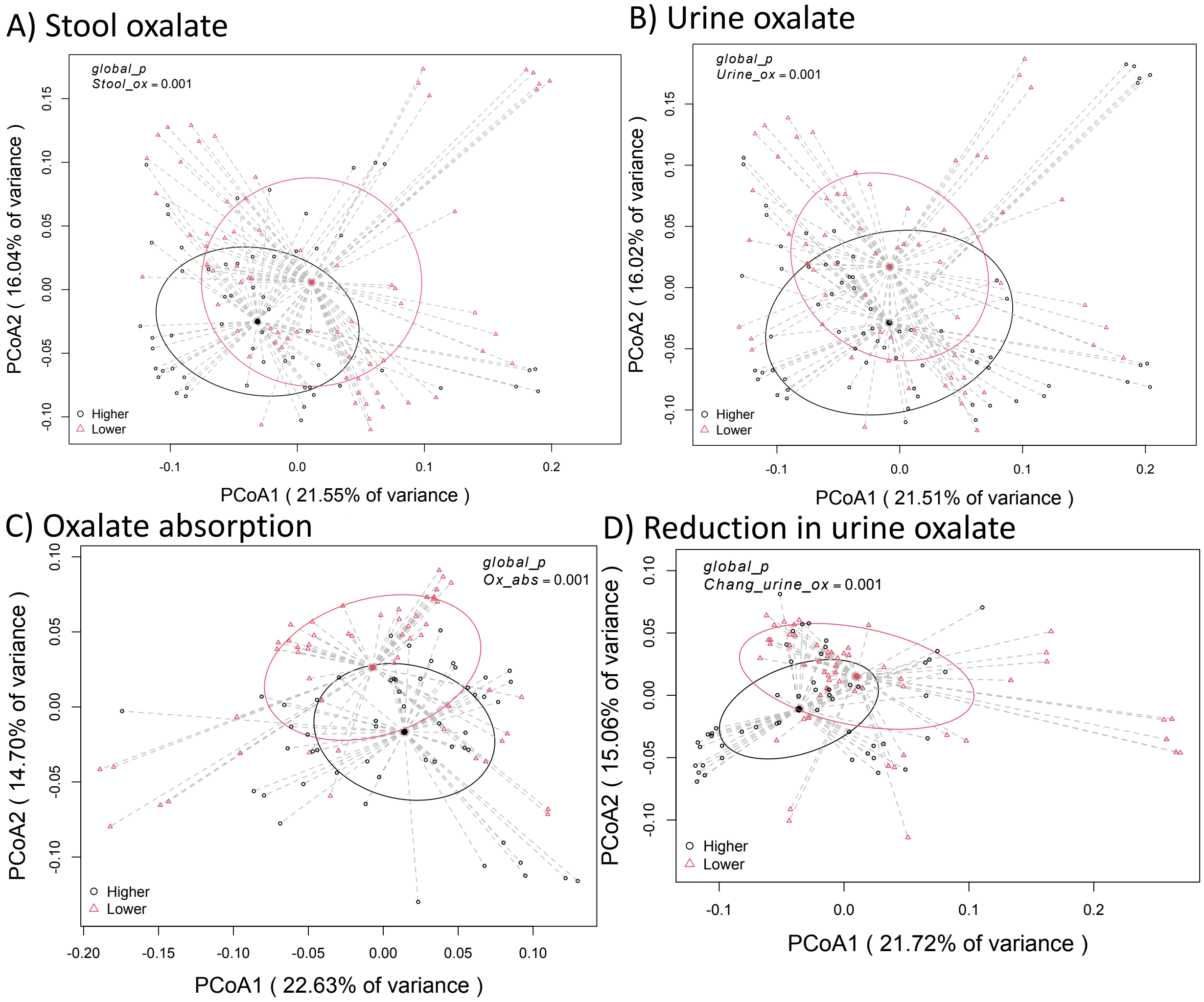
A) Stool oxalate
global_p
Stool_ox = 0.001
Higher
Lower
PCoA1 (21.55% of variance)
PCoA2 (16.04% of variance)
B) Urine oxalate
global_p
Urine_ox = 0.001
Higher
Lower
PCoA1 (21.51% of variance)
PCoA2 (16.02% of variance)
C) Oxalate absorption
global_p
Ox_abs = 0.001
Higher
Lower
PCoA1 (22.63% of variance)
PCoA2 (14.70% of variance)
D) Reduction in urine oxalate
global_p
Chang_urine_ox = 0.001
Higher
Lower
PCoA1 (21.72% of variance)
PCoA2 (15.06% of variance)
